# Epidemiologic History and Genetic Diversity Origins of Chikungunya and Dengue Viruses, Paraguay

**DOI:** 10.3201/eid2705.204244

**Published:** 2021-05

**Authors:** Tiago Gräf, Cynthia Vazquez, Marta Giovanetti, Fernanda de Bruycker-Nogueira, Vagner Fonseca, Ingra Morales Claro, Jaqueline Goes de Jesus, Andrea Gómez, Joilson Xavier, Marcos Cesar Lima de Mendonça, Shirley Villalba, Juan Torales, Maria Liz Gamarra, Julien Thézé, Ana Maria Bispo de Filippis, Vasco Azevedo, Tulio de Oliveira, Leticia Franco, Carlos F. Campelo de Albuquerque, Sandra Irala, Edward Charles Holmes, Jairo Andrés Méndez Rico, Luiz Carlos Junior Alcantara

**Affiliations:** Fundação Oswaldo Cruz, Salvador, Brazil (T. Gräf, J.G. de Jesus);; Fundação Oswaldo Cruz, Rio de Janeiro, Brazil (C. Vazquez, M. Giovanetti, F. de Bruycker-Nogueira, V. Fonseca, J. Xavier, M.C.L. de Mendonça, A.M. Bispo de Filippis, L.C.J. Alcantara);; Laboratorio Central de Salud Pública, Asunción, Paraguay (C. Vazquez, A. Gómez, S. Villalba, J. Torales, M.L. Gamarra);; Instituto de Ciências Biológicas, Universidade Federal de Minas Gerais, Belo Horizonte, Brazil (M. Giovanetti, V. Fonseca, J. Xavier, V. Azevedo, L.C.J. Alcantara);; University of KwaZulu-Natal, Durban, South Africa (V. Fonseca, T. de Oliveira);; Universidade de São Paulo, São Paulo, Brazil (I.M. Claro);; University of Oxford, Oxford, UK (J. Thézé);; University of Washington, Seattle, Washington, USA (T. de Oliveira);; Organización Panamericana de la Salud /Organización Mundial de la Salud, Panama City, Panama (L. Franco);; Organização Pan-Americana da Saúde/Organização Mundial da Saúde, Brasília, Brazil (C.F. Campelo de Albuquerque);; Dirección General de Vigilancia de la Salud Paraguay, Asunción (S. Irala);; The University of Sydney, Sydney, New South Wales, Australia E.C. Holmes);; Pan American Health Organization/World Health Organization, Washington, DC, USA (J.A.M. Rico)

**Keywords:** genomic surveillance, chikungunya virus, dengue virus, nanopore sequencing, arboviruses, viruses, vector-borne infections, Paraguay

## Abstract

Paraguay has been severely affected by emergent Zika and chikungunya viruses, and dengue virus is endemic. To learn more about the origins of genetic diversity and epidemiologic history of these viruses in Paraguay, we deployed portable sequencing technologies to strengthen genomic surveillance and determine the evolutionary and epidemic history of arthropod-borne viruses (arboviruses). Samples stored at the Paraguay National Central Laboratory were sequenced and subjected to phylogenetic analysis. Among 33 virus genomes generated, we identified 2 genotypes of chikungunya and 2 serotypes of dengue virus that circulated in Paraguay during 2014–2018; the main source of these virus lineages was estimated to be Brazil. The evolutionary history inferred by our analyses precisely matched the available travel history of the patients. The genomic surveillance approach used was valuable for describing the epidemiologic history of arboviruses and can be used to determine the origins and evolution of future arbovirus outbreaks.

Chikungunya virus (CHIKV), dengue virus (DENV), and Zika virus (ZIKV) are 3 of the most common arthropod-borne viruses (arboviruses) that infect humans. All are transmitted by the anthropophilic and urban-adapted *Aedes aegypti* and *Ae. albopictus* mosquito vectors (*1*). Driven by human movement and climate trends, the distribution of these mosquitoes is expanding along with the arboviruses they transmit (*2*). In Latin America, CHIKV and ZIKV have emerged since the mid-2000s, joining DENV, which is already endemic there (*3*). In this region, only Uruguay and Chile did not report autochthonous transmissions of one of these arboviruses during 2014–2019, highlighting the current state of endemicity (*4*).

Paraguay is a landlocked country in the center of South America; it borders Bolivia, Brazil, and Argentina. DENV is endemic to Paraguay, and all 4 serotypes (DENV-1–4) have been detected there; in some seasons, multiple serotypes co-circulate (*5*,*6*). Phylogenetic analysis has shown that DENV genetic diversity in Paraguay is closely related to that in neighboring countries, particularly Brazil (*7*,*8*). However, more genomic surveillance studies in Paraguay are needed to learn more about this epidemiologic pattern. Cases of chikungunya fever in Paraguay were first reported in June 2014; autochthonous cases were first detected in 2015, and CHIKV caused seasonal outbreaks every year until 2018. Zika was first detected in November 2015, and autochthonous infections were confirmed soon after (*9*). To date, however, little is known about the genetic diversity of CHIKV and ZIKV that circulate in Paraguay.

The potential triple epidemic scenario (i.e., CHIKV, DENV, ZIKV) in Paraguay could pose serious public health and economic burdens. Arbovirus surveillance is critical for assisting health services with preparedness, providing key information about the seasonality of infections and diversity of circulating viral lineages. When resources allow, such surveillance can now involve genomic surveillance via portable sequencing technologies. For example, this approach was successfully used to study the ZIKV epidemic in the Americas (*10*,*11*), the reemergence of yellow fever virus in Brazil (*12*,*13*), and recurrent outbreaks of CHIKV in several regions of Brazil (*14**–**16*).

To help reinforce arbovirus surveillance in Paraguay, we performed portable genome sequencing under the scope of the ZIBRA project (http://zibraproject.org) at the Laboratorio Central de Salud Publica in Asunción, Paraguay. During July 16–20, 2018, a team of molecular biologists from Brazil and Paraguay worked on a group of samples selected to determine the recent history of arboviruses in the country, generating 33 viral genomes and building capacity skills among the local laboratory staff. We report the analysis of the origins and spread of CHIKV in Paraguay as well as the current dynamics of DENV. The project was reviewed and approved by the Comissão Nacional de Ética em Pesquisa (CONEP) from the Brazilian Ministry of Health as part of the arbovirus genomic surveillance efforts within the terms of CONEP Resolution 510/2016 by the Pan American Health Organization Ethics Review Committee (PAHO-2016-08-0029) and by the Paraguayan Ministry of Public Health and Social Welfare (MSPyBS/S.G. no. 0944/18).

## Methods

### Sample and Data Collection

This study was necessarily based on convenience sampling; de-identified samples were obtained from material exceeding the routine number of arbovirus diagnoses and stored at the Laboratorio Central de Salud Publica de Asunción, Paraguay, which concentrates biological samples collected throughout the country. On the basis of resources and time availability, we selected 50 acute-phase serum samples that were positive for DENV or CHIKV with PCR cycle threshold (C_t_) values <28 at the time of diagnosis. Using epidemiologic data, we chose samples to represent geographic departments in Paraguay with the highest number of cases. ZIKV-positive samples with low C_t_ and historical samples for DENV were unavailable; hence, for DENV, we studied only the 2018 epidemic. The Direccion General de Vigilancia de la Salud of Paraguay provided temporal data on the incidence of chikungunya and dengue cases by department within Paraguay.

### Virus Amplification and Whole-Genome Sequencing

We extracted viral RNA from the selected samples by using the QIAamp Viral RNA Mini Kit (QIAGEN, https://www.qiagen.com) and subjected the RNA to real-time reverse transcription quantitative PCR to detect CHIKV and DENV serotypes 1–4 as described previously (*17**–**19*). To increase the genome coverage, we selected only samples with C_t_ <35 for sequencing. Extracted RNA was converted to cDNA by using the Protoscript II First Strand cDNA Synthesis Kit (New England Biolabs, Inc., https://www.neb.uk.com) and random hexamer priming. We attempted whole-genome amplification by multiplex PCR as previously described (*20*).

We purified amplicons by using 1x AMPure XP Beads (Beckman Coulter, https://www.beckman.com) and quantified them on a Qubit 3.0 fluorimeter by using a Qubit dsDNA HS Assay Kit (ThermoFisher Scientific, https://www.thermofisher.com). We performed DNA library preparation by using a Ligation Sequencing Kit and Native Barcoding Kit (NBD103; Oxford Nanopore Technologies, https://nanoporetech.com). We generated sequencing libraries from the barcoded products by using the Genomic DNA Sequencing Kit SQK-MAP007/SQK-LSK208 and loaded them into an R9.4 flow cell (Oxford Nanopore Technologies).

### Generation of Consensus Sequences

We base-called raw files by using Albacore software, demultiplexed and trimmed by using Porechop software (https://github.com) and then mapped with Burrows-Wheeler Aligner software to a reference genome. On the basis of PCR analyses, we used GenBank accession nos. KP164568 for CHIKV, KF672760 for DENV-1, and JN559741 for DENV-4 as reference sequences. To detect single-nucleotide variants to the reference genome, we applied Nanopolish software variant calling (https://nanoporetech.com) to the assembly. Nonoverlapped primer binding sites and sites for which coverage was <20× were replaced with ambiguity code N.

### Phylogenetic Analyses

**Figure 1:**
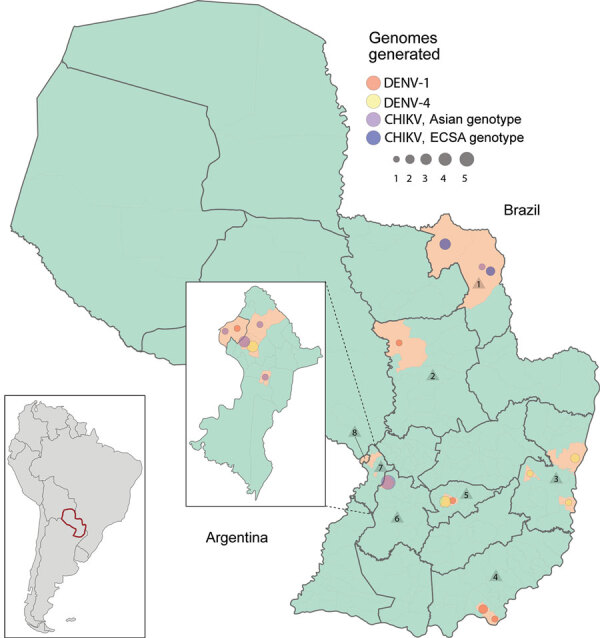
Geopolitical map of Paraguay showing locations of sampling for dengue virus (DENV) and chikungunya virus (CHIKV). Circle sizes are scaled to represent the number of genomes isolated in each municipality. Numbers inside triangles indicate sampled departments: 1, Amambay; 2, San Pedro; 3, Alto Paraná; 4, Itapúa; 5, Guairá; 6, Paraguarí; 7, Central; 8, Asunción. Callout map shows the Central and Asunción Departments of Paraguay; inset map shows the location of Paraguay in South America.

We first investigated sequence genotypes by using the arbovirus genotyping tool (*21*). To investigate the origins and spatial dynamics of arboviruses in Paraguay, we downloaded all sequences assigned as CHIKV, DENV-1, and DENV-4 from GenBank. We excluded sequences without sampling date and location and sequences covering <50% of the virus genome. Sequence alignment was performed by using MAFFT (*22*) (FFT-NS-2 algorithm) and visually inspected in Aliview (*23*). We estimated maximum-likelihood phylogenies in IQ-TREE (*24*) by using the best-fit model of nucleotide substitution as indicated by the ModelFinder application (implemented in IQ-TREE). Branch support was assessed by the SH-like approximate-likelihood ratio test, and we submitted highly supported (>0.9) clades containing the DENV genomes from Paraguay (Appendix 1 Figure 1) and the clades of CHIKV from the Americas to TempEst (*25*) to assess the strength of temporal signal in these data.

Time-scaled phylogenetic trees were inferred by using the BEAST package (*26*). We chose the uncorrelated relaxed molecular clock model as indicated by the marginal likelihood estimation model test procedure. We also used the codon-based SRD06 model of nucleotide substitution and the nonparametric Bayesian Skygrid coalescent model. A discrete phylogeographical model (*27*) was used to reconstruct the spatial diffusion of the virus across the compiled dataset sampling locations (Appendix 2). Phylogeographic analyses were performed by applying an asymmetric model of location transitioning coupled with the Bayesian stochastic search variable selection procedure. We complemented this analysis with Markov jump estimation that counts location transitions per unit time along the tree. We ran Monte Carlo Markov chains long enough to ensure stationarity and an adequate effective sample size of >200.

## Results

**Figure 2:**
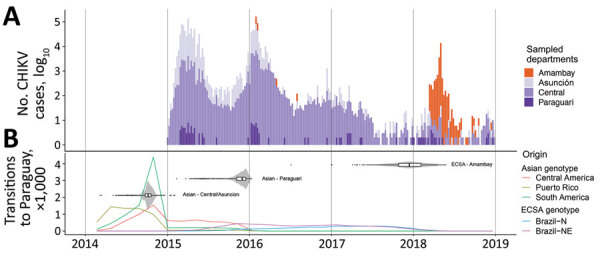
Chikungunya virus (CHIKV) outbreaks in Paraguay during 2014–2018 and the spatial–temporal history of virus diffusion. A) Total number of cases of CHIKV infection reported by epidemiologic week in the departments from which genome sequences were available. B) Location transitions to Paraguay inferred from the posterior distribution of phylogenetic trees by the Markov jump approach, and the time to most recent common ancestor for the CHIKV clusters detected in the country. Lines are colored according to the origin of the estimated transition toward Paraguay. Violin plots show 95% CIs with internal boxplots showing median and interquartile ranges. Brazil-N, Brazil North Region; Brazil-NE, Brazil Northeast Region; ECSA, East/Central/South African genotype.

Of the 50 samples tested, 25 were positive for CHIKV, 14 for DENV-1, and 11 for DENV-4. For positive samples, the average PCR C_t_ value was 26.36 (range 16–37). From the 50 samples, we were able to generate 33 complete or near-complete genome sequences (17 CHIKV and 16 DENV genomes) (Table). The GenBank accession numbers of newly generated sequences are MT038393–409 (CHIKV) and MT040672–87 (DENV). The collection dates of the samples sequenced were November 3, 2014, through July 10, 2018, and locations covered 15 municipalities and 8 departments (the first-level administrative subdivisions) of Paraguay (Figure 1). Women accounted for 58% of the samples, and the median patient age was 34 years. A TempEst analysis of all arbovirus lineages found here revealed a strong correlation between the sampling time and the root-to-tip divergence (Appendix 1 Figure 2).

**Table:** Patient demographic and virus sequencing data for samples from the Laboratorio Central de Salud Publica de Asunción, Paraguay, 2014–2018*

Sample	Virus	Department, municipality	Collection date	Patient age, y/sex	C_t_	Reads	Genome coverage, %
PY02	CHIKV-Asian genotype	Paraguarí, Yaguarón	2016 Jan 20	40/M	23.6	204,763	88.1
PY03	CHIKV-Asian genotype	Paraguarí, Yaguarón	2016 Jan 21	67/M	16.9	215,137	87.0
PY06	CHIKV-Asian genotype	Paraguarí, Yaguarón	2016 Feb 16	9/M	26.9	282,182	88.3
PY07	CHIKV-Asian genotype	Paraguarí, Yaguarón	2016 Fab 19	34/F	29.7	267,784	87.2
PY08	CHIKV-Asian genotype	Paraguarí, Yaguarón	2016 Feb 22	39/F	30.2	142,555	87.1
PY09	CHIKV-Asian genotype	Asunción, Asunción	2016 Mar 21	27/F	28	265,596	87.9
PY12	CHIKV-ECSA/BR	Amambay, Pedro Juan Caballero	2018	21/M	29	236,285	84.8
PY13	CHIKV-ECSA/BR	Amambay, Pedro Juan Caballero	2018 Jun 25	35/F	28	175,112	85.3
PY15	CHIKV-ECSA/BR	Amambay, Bella Vista Norte	2018 Jun 29	40/F	34	17,030	70.9
PY17	CHIKV-ECSA/BR	Amambay, Bella Vista Norte	2018 Jul 3	22/M	34	320,142	86.8
PY18	CHIKV-ECSA/BR	Amambay, Bella Vista Norte	2018 Jul 10	57/M	29	315,588	86.0
PY19	DENV-4	Guairá, Villarrica	2018 Apr 23	38/F	23	22,041	82.3
PY21	DENV-4	Guairá, Villarrica	2018 Apr 26	68/F	22	21,042	96.0
PY22	DENV-4	Guairá, Villarrica	2018 Apr 27	52/M	22	13,213	96.0
PY23	DENV-4	Central, San Lorenzo	2018 May 6	19/M	27	11,548	74.6
PY24	DENV-1	San Pedro, San Pedro De Ycuamandyju	2018 May 4	29/F	20	7,265	89.1
PY25	DENV-4	Central, San Lorenzo	2018 May 5	38/F	21	17,299	96.0
PY27	DENV-4	Alto Paraná, Domingo Martinez De Irala	2018 May 9	27/F	19	21,188	96.0
PY28	DENV-4	Alto Paraná, Hernandarias	2018 May	30/M	21	22,800	86.6
PY31	DENV-4	Alto Paraná, Hernandarias	2018 May 22	14/M	32	8,770	95.9
PY32	DENV-4	Central, San Lorenzo	2018 May 31	28/M	28	6,907	96.0
PY33	DENV-1	Asunción, Asunción	2018 May 31	3/F	22	9,846	76.8
PY34	DENV-4	Alto Paraná, Juan Leon Mallorquin	2018 May 28	47/F	26	7,945	96.0
PY35	DENV-1	Itapúa, Encarnacion	2018 Jun 7	62/F	23	119,293	89.1
PY36	DENV-1	Itapúa, Encarnacion	2018 Jun 8	61/M	23	6,448	76.8
PY38	DENV-1	Itapúa, Cambyreta	2018 Jun 8	6/F	25	111,057	89.1
PY43	DENV-1	Guairá, Villarrica	2018 Jun 4	53/F	25	8,779	89.1
PY44	CHIKV-Asian genotype	Central, Luque	2014 Nov 30	33/F	24	13,687	85.1
PY45	CHIKV-Asian genotype	Amambay, Pedro Juan Caballero	2014 Nov 3	54/M	24	12,214	86.6
PY47	CHIKV-Asian genotype	Central, Guarambare	2015 Apr 26	25/M	17	9,536	85.2
PY48	CHIKV-Asian genotype	Central, Fernando De La Mora	2015 Apr 28	50/F	18	6,002	86.6
PY49	CHIKV-Asian genotype	Central, Fernando De La Mora	2015 May 3	12/F	27	53,928	86.4
PY50	CHIKV-Asian genotype	Central, Fernando De La Mora	2015 May 6	25/F	16	49,813	86.8

*CHIKV, chikungunya virus; C_t_, cycle threshold; DENV, dengue virus; ECSA/BR, East/Central/South African genotype from Brazil.

### The CHIKV Epidemic in Paraguay during 2014–2018

**Figure 3:**
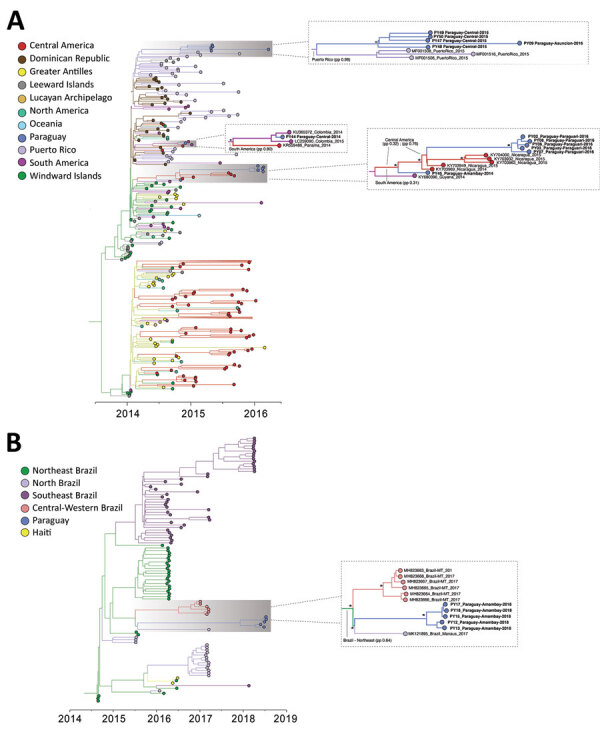
Time-scaled phylogenetic trees of chikungunya virus (CHIKV) genomes isolated in the Americas. A) CHIKV Asian genotype; B) CHIKV East/Central/South African genotype from Brazil. Tips and internal branches are colored according to the most likely geographic location, and ancestral states were estimated by phylogeographic methods. Clusters relevant to the epidemic in Paraguay are shown in detail where the most likely ancestral state estimation is annotated. Asterisks indicate highly supported clusters (posterior probability >0.9).

Of the 17 CHIKV genomes, 12 were classified as Asian genotype (sampled during 2014–2016) and 5 as East/Central/South African (ECSA) genotype (sampled during 2018). The oldest CHIKV sample analyzed (patient PY45) was obtained in November 2014 from the department of Amambay and was identified as an Asian genotype (Figure 1). However, autochthonous transmission of CHIKV was not confirmed until February 2015, followed by an increased number of reported infections (*9*) (Figure 2, panel A). Phylogeographic analysis revealed that the most likely origin of PY45 was Central or South America (Figure 3, panel A). The travel history for PY45 is in agreement with these results because the patient reported having visited Panama and San Andrés, a Colombian cluster of islands in the Caribbean region. In November 2014, another introduction of CHIKV in Paraguay was detected in the Central Department. That sequence (patient PY44) clustered with high support (posterior probability [PP] = 1) among sequences from Colombia and 1 sequence from Nicaragua, and the ancestral state of the most recent common ancestor (MRCA) of this clade was South America (PP = 0.8). Patient PY44 reported traveling to Cartagena, Colombia, supporting the origin estimated by the phylogeographic analysis.

In 2015, a large CHIKV epidemic occurred in Paraguay, resulting in ≈10,000 cases (suspected and confirmed); the main affected departments were Central, Asunción, and Paraguarí (Figure 2, panel A). All genomes generated from the 4 samples from 2015 were classified as members of the Asian genotype and grouped together with high support (PP = 1) (Figure 3, panel A) in a clade for which time to MRCA (tMRCA) was October 2014 (95% highest posterior density [HPD] May 2014 to November 2014) (Figure 2, panel B). From our analysis, we estimated that the geographic origin of the variant circulating in Paraguay in 2015 was Puerto Rico (PP = 0.98) (Figure 3, panel A). The first patient with autochthonous CHIKV infection in Paraguay is believed to be the housemaid of a family returning from Puerto Rico in October 2014, who sought healthcare services for symptoms of chikungunya fever. CHIKV infections were confirmed for the housemaid and the family, and our phylogenetic analysis confirmed this epidemiologic history.

The time distributions of CHIKV infection cases from 2016 were very similar to those in 2015 (Figure 2, panel A), although they did reveal that at least 2 lineages were circulating in the country (Figure 3, panel A). A new introduction is likely to have occurred in the Paraguarí Department at the mean time point of November 2015 (95% HPD June 2015 to January 2016); the most likely place of origin was Central America (PP = 0.76) (Figure 3, panel A). However, the sequence from Amambay Department, isolated in 2014, is positioned basally to the Paraguarí cluster, suggesting that the same variant persisted in the country up to 2016. Year-round persistence of a CHIKV strain is clearly observed in the Central/Asunción Department cluster in which the genome isolated in 2016 clustered with the genomes isolated in 2015 (Figure 3, panel A).

Besides the Asian genotype of CHIKV, an outbreak of the ECSA genotype occurred in Paraguay in 2018. After a year of very few CHIKV infections in 2017 (Figure 2, panel A), a new outbreak was observed in 2018 (although milder than that of 2015 and 2016), and the main affected department was Amambay. Five genomes from this department revealed circulation of the CHIKV ECSA genotype in 2018, and the mean tMRCA of this cluster was December 2017 (95% HPD July 2017 to April 2018) (Figure 2, panel B). The source of introduction of this new CHIKV lineage in Paraguay was estimated to be Brazil, most likely the Northeast Region (PP = 0.64), or perhaps the North Region (PP = 0.32) (Figure 3, panel B).

We also summarized all geographic locations that had significantly (Bayes factor >3) seeded new CHIKV lineages to Paraguay and superimposed it onto the tMRCA of the 3 main CHIKV clusters detected there (Figure 2, panel B). Most transitions in the Asian genotype occurred in 2014, when CHIKV was spreading rapidly through the Americas. Far fewer transitions were estimated to have occurred in 2015, which accords with the hypothesis that the 2016 outbreak in Paraguarí was caused by a lineage already circulating in the country. For the ECSA genotype of CHIKV, the importations from Brazil were widespread between the middle of 2015 and the beginning of 2019. These widespread importations result from the long branch connecting the Paraguay ECSA cluster to the Brazilian sequences (Figure 3, panel B), increasing uncertainty in the relevant parameter estimates.

### Genetic Diversity of DENV in 2018

**Figure 4:**
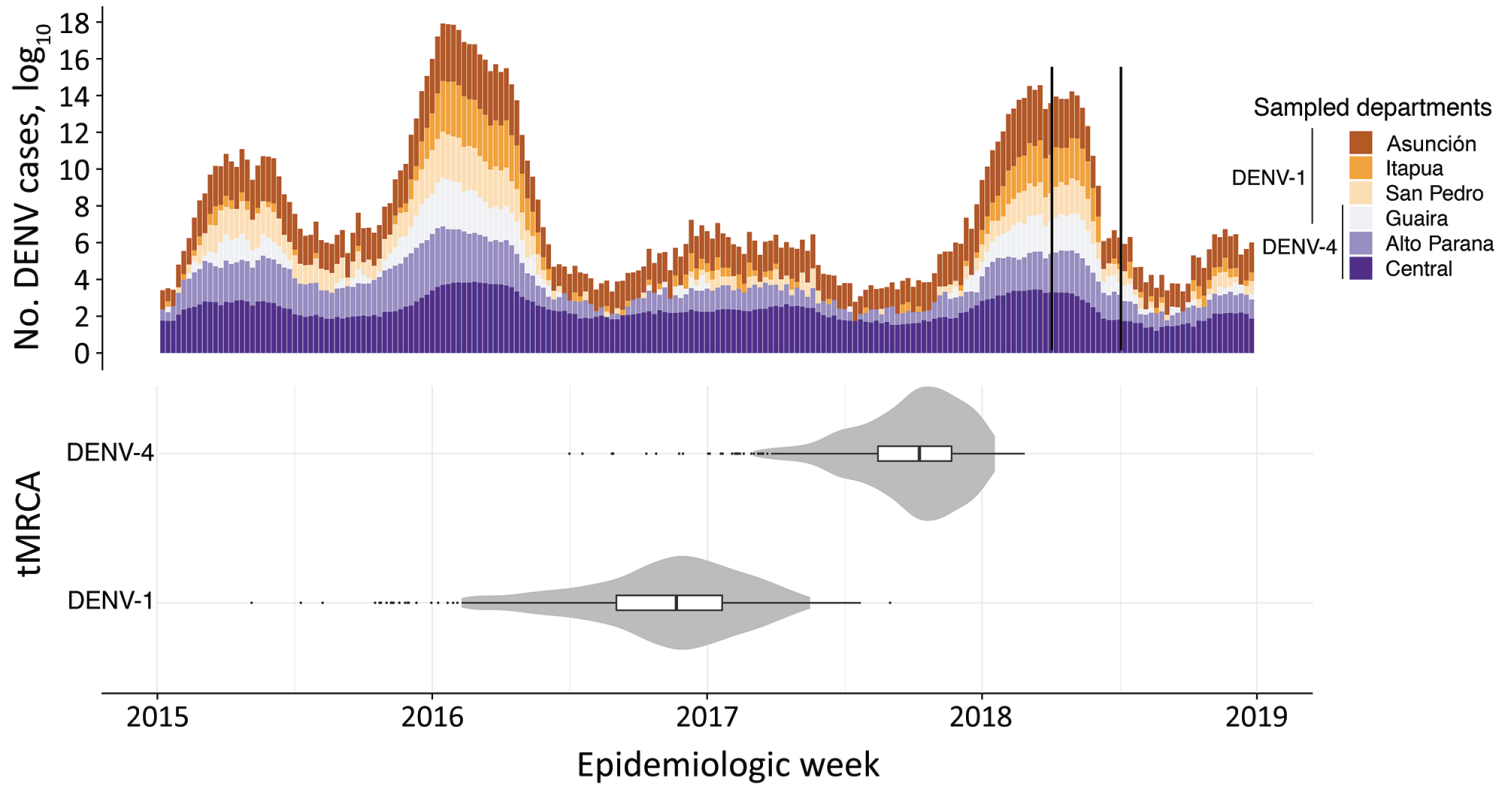
Dengue virus (DENV) outbreaks in Paraguay during 2015–2018 and tMCRA of serotypes 1 and 4. A) Total cases of DENV infections reported by epidemiologic week in the departments from which genome sequences were available. The black bars in 2018 delimit the sampling time range for the DENV genomes. B) tMRCA for DENV-1 and DENV-4 in the same timescale as the number of cases reported. Violin plots show 95% CIs; internal boxplots show medians and interquartile ranges. tMCRA, time to most recent common ancestor.

The number of DENV cases in Paraguay during 2015–2018 shows a very similar pattern to that for CHIKV (Figure 2, panel A; Figure 4, panel A). Case numbers were higher at the beginning of each year, except for 2017 when the DENV season was atypically mild. Our sampling from 2018 captured the 2 DENV serotypes (DENV-1 and DENV-4) circulating in the country (*9*), and molecular clock analyses estimated that DENV-4 was introduced in Paraguay just before the beginning of the 2018 outbreak, whereas DENV-1 was already circulating in 2017 (Figure 4, panel B).

**Figure 5:**
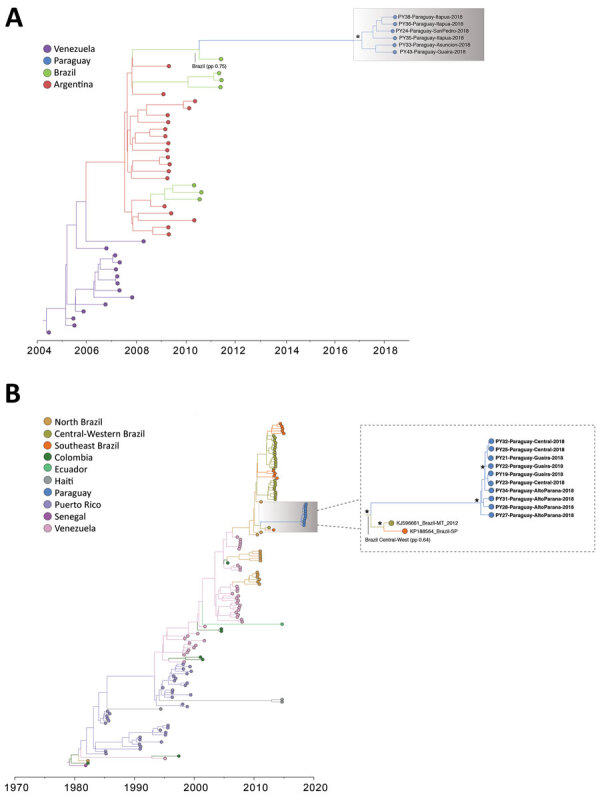
Time-scaled phylogenetic trees of dengue virus (DENV) serotypes 1 and 4 genomes isolated in Paraguay and related sequences. A) DENV-1; B) DENV-4. Tips and internal branches are colored according to geographic location, and ancestral states were estimated by phylogeographic methods. Clusters relevant to the epidemic in Paraguay are shown in detail where the most likely ancestral state estimation is annotated. Asterisks indicate highly supported clusters (posterior probability >0.9).

DENV-1 and DENV-4 sequences from Paraguay clustered together with high support (PP>0.9) and belonged to genotypes V and II, respectively, which predominate in Latin America (Figure 5). The most likely origin of the DENV-1 strain circulating in Paraguay in 2018 was estimated to be Brazil (PP = 0.75) (Figure 5, panel A), and the mean tMRCA was estimated to be October 2016 (95% HPD February 2016 to May 2017) (Figure 4, panel B). DENV-4 was also estimated to have an origin in Brazil; the best-supported regions of origin were Central-West (PP = 0.64) and North (PP = 0.34) (Figure 5, panel B). The mean tMRCA of DENV-4 was September 2017 (95% HPD April 2017 to February 2018), ≈1 year later than DENV-1. Examining the DENV-4 cluster in Paraguay in more detail revealed that sequences from the Alto Paraná Department are basal and that sequences from the Central and Guairá Departments group together in a highly supported (PP>0.9) and distal cluster (Figure 5, panel B). Alto Paraná borders Brazil, and although not formally tested because of the small number of sequences, Alto Paraná could be the point of introduction of the current DENV-4 lineage into Paraguay.

## Discussion

The first CHIKV outbreaks in the Americas (the Asian genotype) were reported for the French Caribbean islands of Saint Martin and Martinique in December 2013 (*28*). The virus rapidly spread throughout the Caribbean and Central America in 2014, and autochthonous transmissions were reported in almost all countries/territories of these regions. In 2014, Paraguay reported imported cases only, mostly in persons with a history of travel to Central America or the Caribbean. Our analysis of virus sequences from 2 of these persons with imported cases, and the virus phylogenetic relatedness to foreign viruses, matched the travel history with precision. In addition, the estimated origin of the first outbreak of CHIKV in Paraguay in 2015 agreed both in time (October 2015) and location (Puerto Rico) with the travel data collected by the Paraguay surveillance system.

The CHIKV epidemic in Paraguay in 2016 was very similar to that in 2015, when the most affected departments were Central, Asunción, and Paraguarí. These neighboring departments are located in the most densely populated region of Paraguay, which might lead to consecutive outbreaks. In our sampling, most sequences from 2016 were from the Paraguarí Department and formed a separate clade from the 2015 epidemic, suggesting a new introduction event. However, because of the co-clustering of sequences from the Asunción and Central Departments isolated in 2015 and 2016, we cannot exclude year-round persistence of the virus. Models of *Aedes* spp. mosquito competence for DENV transmission have shown that the Paraguay climate is conducive to year-round persistence (*29*).

In Brazil, 2 distinct lineages of CHIKV were detected at the end of 2014, the ECSA genotype in the Northeast Region and the Asian genotype in the North Region (*30*). Whereas the CHIKV outbreak of the Asian genotype remained restricted to a small number of cases, the ECSA lineage spread throughout Brazil. In this study, we determined that the ECSA genotype was the causative agent of a CHIKV outbreak in Amambay (a Paraguay department on the Brazil border) in 2018. To our knowledge, these are the only 2 countries in the Americas to report outbreaks of both the Asian and ECSA genotypes to date. Our analysis revealed a mean time of entry into Paraguay of around December 2017, most likely from the Northeast Region in Brazil. However, because of the small number of samples from states in Brazil that border Paraguay, all such inferences of geographic origins should be interpreted with caution. It is possible that previous exposure to the CHIKV Asian genotype may have created some population immunity that restricted ECSA circulation in other parts of Paraguay (e.g., Central and Asunción Departments) in 2018. Unfortunately, no data on CHIKV seroprevalence in Paraguay are available to test this hypothesis. Amambay, on the other hand, reported few CHIKV cases during the epidemics of 2015 and 2016 (Figure 2, panel A), potentially enabling the emergence of the ECSA genotype in 2018.

The 2018 dengue season in Paraguay was dominated by DENV-1, reported in all departments (*9*). Three departments (Central, Alto Paraná, and Guaira) also reported the circulation of DENV-4. Our analysis suggests that the origin of both serotypes in Paraguay is Brazil, supporting findings of previous studies (*31*). Although our sampling was restricted to 2018, we observed that the tMRCA of DENV-1 was much earlier (October 2016), suggesting that this lineage may have persisted during the 2017 and 2018 seasons, when it may have been responsible for most cases (*9*). DENV-4, on the other hand, was introduced in September 2017, just before the start of the dengue season, and was responsible for few infections until the 2019–20 season, when it dominated the epidemic (*32*, *33*).

Despite screening all publicly available (GenBank) sequences of DENV from the Americas, we found that the DENV datasets were sparsely distributed in time (DENV-4) or contained large temporal gaps (DENV-1) (Appendix 1 Figure 2), potentially biasing our results. The paucity of available DENV complete genomes in South America constrains the applicability of phylogenetic tools for studying virus population dynamics. It also highlights the value of intensifying sequencing efforts in line with the genomic surveillance approach and for real-time generating and sharing of data. The CHIKV datasets were much more comprehensive; for instance, the Asian lineage dataset analyzed included 291 genomes sampled during 2014–2018, representing 38 countries/territories (Appendix 2). The more comprehensive CHIKV datasets reflect the fact that CHIKV emerged in the Americas in the era of next-generation sequencing, when the development of numerous platforms reduced the cost and shortened the time from sample preparation to data generation. Hence, this increased availability of CHIKV virus genomes allowed a more detailed analysis of the virus’s spatiotemporal history in Paraguay. However, another study limitation is the convenience sampling used, with a narrow breadth, potentially biasing molecular clock dating and location ancestral reconstruction. The limited availability of stored samples from years before 2018, and their possibly limited RNA integrity, impaired genome sequencing from previous outbreaks. Nevertheless, we suggest that the 33 genomes generated here are representative of the current DENV diversity and the recent CHIKV evolutionary history in Paraguay.

Of note, the mean tMRCAs for the 3 clusters of CHIKV and the 2 clusters of DENV were estimated in the last trimester of the year (September–December), the start of arbovirus season in many tropical and subtropical regions in the Southern Hemisphere (e.g., Paraguay). A previous study (*34*) has modeled the timing and scale of arbovirus transmission potential and found that in many cities in Brazil with climates similar to that of Paraguay, transmission starts to increase around September. Thus, the tMRCAs estimated here probably reflect the onset of the arbovirus season, which peaks during January–March. Oddly, the 2017 season was marked by a noticeable reduction in cases of both DENV and CHIKV in Paraguay (Figure 2, panel A; Figure 5, panel A). This pattern was observed for dengue throughout the Americas, where cases decreased by 73% in 2017 compared with 2016 (*35*). It is possible that the mild season in 2017 might be explained by a transient strengthening of vector control interventions, implemented after the arrival of CHIKV and ZIKV in the Americas and the consequent public health emergency triggered by these pathogens. In addition, cross-immunity between ZIKV and DENV has been observed in the laboratory (*36*). Hence, population immunity to ZIKV after the 2015–2016 epidemic may have provided some transitory protection against DENV, resulting in lower incidence in 2017 (*37*,*38*), although this protection alone would not explain the decreased CHIKV cases in 2017 in Paraguay.

In conclusion, our study reveals a complex pattern of arbovirus circulation in Paraguay. We identify Brazil as a source of CHIKV and DENV lineages and show that other countries from South America and the Caribbean, mainly tourist destinations, were also hubs of virus spread toward Paraguay. Our sequencing and phylogenetic analyses proved to be powerful tools for revealing the transmission dynamics between the sampled locations and matched, with striking precision, available patient travel history. With support from the Pan American Health Organization, this project developed capacity-building skills in Paraguay, which can be applied in future arbovirus outbreaks.

Appendix 1 Supplementary figures for study of epidemiologic history and genetic diversity origins of dengue and chikungunya viruses, Paraguay.

Appendix 2 Supplementary tables for study of epidemiologic history and genetic diversity origins of dengue and chikungunya viruses, Paraguay.
