# Supplementary material for: Epidemiologic History and Genetic Diversity Origins of Chikungunya and Dengue Viruses, Paraguay
Source: Emerg Infect Dis. 2021 May;27(5):1393–404. doi: 10.3201/eid2705.204244 (PMC8084490; doi:10.3201/eid2705.204244)
Supplement: Appendix 1 — Supplementary figures for study of epidemiologic history and genetic diversity origins of dengue and chikungunya viruses, Paraguay. [file 20-4244-Techapp-s1.pdf]

Article DOI: https://doi.org/10.3201/eid2705.204244

# Epidemiologic History and Genetic Diversity Origins of Chikungunya and Dengue Viruses, Paraguay

## Appendix 1

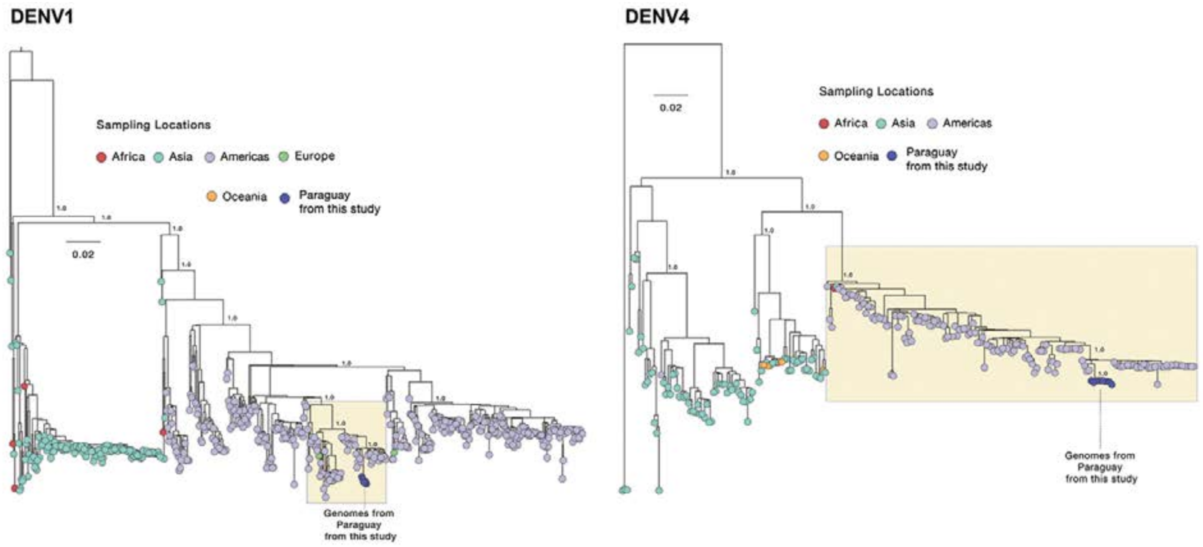


**Appendix Figure 1.** Maximum likelihood phylogenetic tree with DENV1 (left) and DENV4 (right) complete genomes. Tips' circles are colored according to the sampling continent and Paraguayan genomes generated in this study are shown. Highlighted clades were submitted to Bayesian phylogenetic analysis.

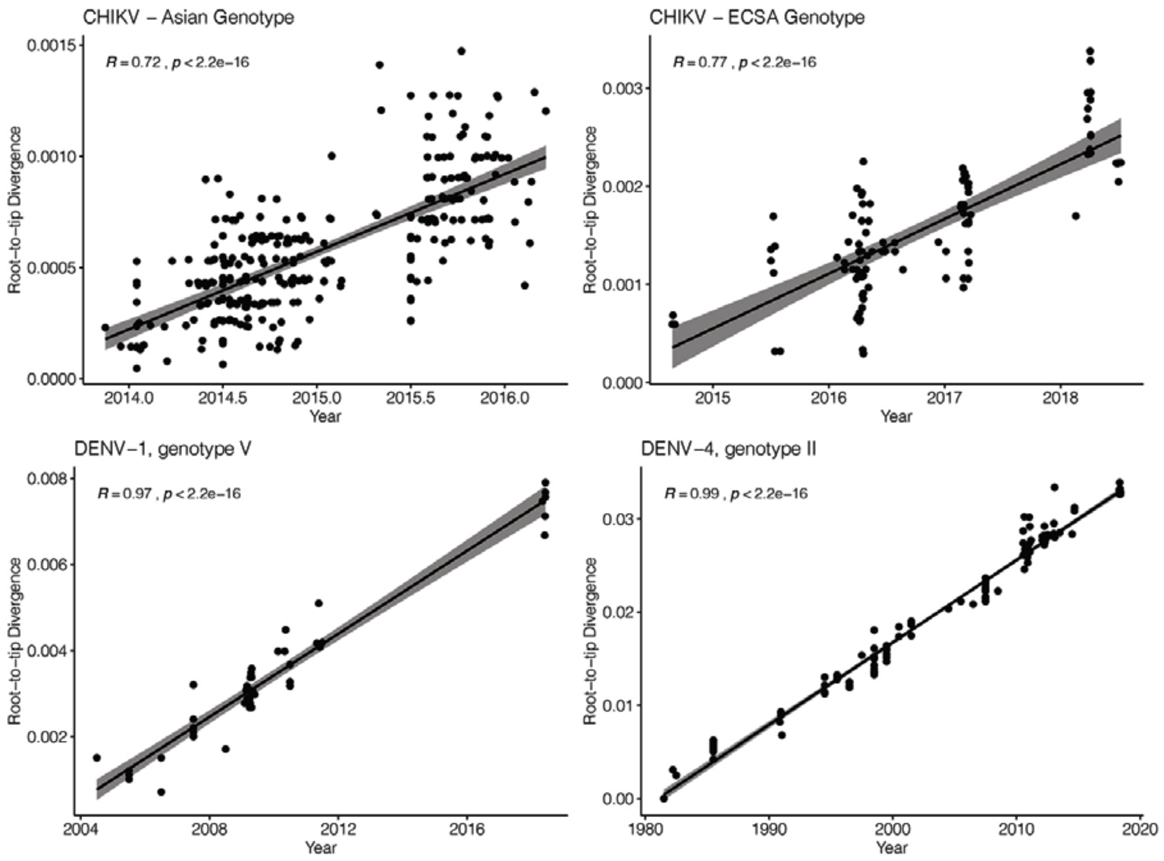


**Appendix Figure 2.** Regression analysis of the root-to-tip divergence against sampling date for the four viral lineages found in Paraguay in this study.
